# Factors and predictive model associated with perioperative complications after long fusion in the treatment of adult non-degenerative scoliosis

**DOI:** 10.1186/s12891-021-04361-y

**Published:** 2021-05-25

**Authors:** Nan Wu, Jiashen Shao, Zhen Zhang, Shengru Wang, Ziquan Li, Sen Zhao, Yang Yang, Lian Liu, Chenxi Yu, Sen Liu, Zhengye Zhao, You Du, Yuanqiang Zhang, Lianlei Wang, Yu Zhao, Keyi Yu, Hong Zhao, Jianxiong Shen, Guixing Qiu, Deciphering Disorders Involving Scoliosis and COmorbidities (DISCO) study, Guixing Qiu, Nan Wu, Shengru Wang, Jiaqi Liu, Sen Liu, Yuzhi Zuo, Gang Liu, Chenxi Yu, Lian Liu, Jiashen Shao, Sen Zhao, Zihui Yan, Hengqiang Zhao, Yuchen Niu, Xiaoxin Li, Huizi Wang, Congcong Ma, Zefu Chen, Bowen Liu, Xi Cheng, Jiachen Lin, Huakang Du, Yaqi Li, Shuang Song, Weijie Tian, Zhixin Xie, Zhengye Zhao, Lina Zhao, Zhi Zhao, Zhifa Zheng, Yingzhao Huang, Zhihong Wu, Terry Jianguo Zhang

**Affiliations:** 1 Department of Orthopedic Surgery, Peking Union Medical College Hospital, Peking Union Medical College and Chinese Academy of Medical Sciences, Beijing, 100730 China; 2 Key Laboratory of Big Data for Spinal Deformities, Chinese Academy of Medical Sciences, Beijing, 100730 China; 3 Beijing Key Laboratory for Genetic Research of Skeletal Deformity, Beijing, 100730 China; 4 Graduate School of Peking Union Medical College, Beijing, 100005 China; 5 Department of Orthopedic Surgery, Qilu Hospital of Shandong University, Jinan, 250012 China; 6 Medical Research Center, Peking Union Medical College Hospital, Peking Union Medical College and Chinese Academy of Medical Sciences, Beijing, China

**Keywords:** Adult non-degenerative scoliosis, Perioperative complications, Risk factors, Long-segment posterior instrumentation and fusion

## Abstract

**Introduction:**

Adult non-degenerative scoliosis accounts for 90% of spinal deformities in young adults. However, perioperative complications and related risk factors of long posterior instrumentation and fusion for the treatment of adult non-degenerative scoliosis have not been adequately studied.

**Methods:**

We evaluated clinical and radiographical results from 146 patients with adult non-degenerative scoliosis who underwent long posterior instrumentation and fusion. Preoperative clinical data, intraoperative variables, and perioperative radiographic parameters were collected to analyze the risk factors for perioperative complications. Potential and independent risk factors for perioperative complications were evaluated by univariate analysis and logistic regression analysis.

**Results:**

One hundred forty-six adult non-degenerative scoliosis patients were included in our study. There were 23 perioperative complications for 21 (14.4%) patients, eight of which were cardiopulmonary complications, two of which were infection, six of which were neurological complications, three of which were gastrointestinal complications, and four of which were incision-related complication. The independent risk factors for development of total perioperative complications included change in Cobb angle (odds ratio [OR] = 1.085, 95% CI = 1.035 ~ 1.137, *P* = 0.001) and spinal osteotomy (OR = 3.565, 95% CI = 1.039 ~ 12.236, *P* = 0.043). The independent risk factor for minor perioperative complications is change in Cobb angle (OR = 1.092, 95% CI = 1.023 ~ 1.165, *P* = 0.008). The independent risk factors for major perioperative complications are spinal osteotomy (OR = 4.475, 95% CI = 1.960 ~ 20.861, *P* = 0.036) and change in Cobb angle (OR = 1.106, 95% CI = 1.035 ~ 1.182, *P* = 0.003).

**Conclusions:**

Our study indicate that change in Cobb angle and spinal osteotomy are independent risk factors for total perioperative complications after long-segment posterior instrumentation and fusion in adult non-degenerative scoliosis patients. Change in Cobb angle is an independent risk factor for minor perioperative complications. Change in Cobb angle and spinal osteotomy are independent risk factors for major perioperative complications.

## Introduction

Adult spinal deformities (ASD) can be classified into two subtypes: progression of childhood scoliosis (non-degenerative scoliosis) and degenerative scoliosis [1]. Because conservative treatment is often insufficient to effectively improve the diverse symptoms, surgical treatment is usually recommended. When a spinal surgery is advocated, determining the extent of the fusion is important. Long-segment instrumentation and fusion have been proven to be able to correct severe deformities and rotatory subluxations [2]. However, long fusions are also associated with excessive intraoperative blood loss, which contributes to the development of perioperative complications [3].

As life expectancy increases, adult degenerative scoliosis (ADS), or de novo scoliosis, is gaining more attention in the field. Patients with ADS suffer from pain, disability, and neurological symptoms [4]. The estimated incidence of postoperative complications has been reported to range from 16.4% to as high as 80% [5, 6]. Many risk factors for these complications have been reported, including massive intraoperative blood loss (> 2 to 4 L), age, the extent and approach of the surgery, and the presence of more than three comorbidities [7–9].

In contrast to ADS, adult non-degenerative scoliosis remains poorly studied. Non-degenerative factors are presumed to account for as high as 90% of the spinal deformities in young adults [10]. Although surgical correction of ASD is cost-effective and improves the quality-of-life and clinical outcomes for scoliosis patients when compared to the non-operative conservative treatment, it is not risk-free. In addition to being associated with worse clinical outcomes and further difficulties in treatment, perioperative complications can impose a substantial clinical and financial burden on state healthcare [11]. In this study, we aim to retrospectively evaluate the potential risk factors for perioperative complications stemming from the use of long-segment posterior-only instrumentation and fusion in the treatment of adult non-degenerative scoliosis.

## Materials and methods

### Patients

A single center-based, retrospective cohort study was performed on adult patients who had undergone long spinal fusions at the Department of Orthopedic Surgery, Peking Union Medical College Hospital. A total of 146 consecutive patients, who were diagnosed with adult non-degenerative scoliosis and who had undergone long-segment internal fixation and fusion by the conventional midline open posterior approach from January 2012 to July 2018, were selected and reviewed. The experimental protocol was reviewed and approved by the Ethics Committee of Peking Union Medical College Hospital (agreement number: JS-908). Our study was performed in accordance with experimental protocol and the Declaration of Helsinki, and informed consent was obtained from all participants.

Inclusion criteria for this study were operatively treated adult spinal deformity patients with the following conditions: (1) age > 18 years by the time of the surgery, (2) major Cobb angle ≥40°, (3) posterior long-segment internal fixation and fusion (≥ 4 vertebrae), (4) follow-up ≥1 year, (5) complete preoperative and postoperative radiographic data and clinical evaluations, (6) complete medical history, and (7) the patient was diagnosed with idiopathic scoliosis. Exclusion criteria were (1) degenerative or de novo scoliosis (degenerative change without preexisting scoliosis typically manifested in the lumbar spine) and other kinds of secondary spinal deformities (e.g., ankylosing spondylitis, spinal tumor, iatrogenic spinal deformity, and posttraumatic spinal deformity); (2) previous history of lumbar surgery; and (3) anterior instrumentation or non-fusion surgery.

### Medical history and operative data

Baseline characteristics, including age, sex, body mass index (BMI), presenting symptoms, any history of smoking, medication use, previous surgeries, comorbidities, preoperative hemoglobin (Hgb) level, and the length of hospital stay (LOS) were collected. American Society of Anesthesiologists (ASA) grades were evaluated by anesthetists. Fusion levels, distal instrumented and fusion levels, estimated blood loss (EBL), duration of the operation, and the volume of blood transfusions were charted. Perioperative complications were defined as any event for which the patient required a specific intervention or treatment. All complications that occurred before discharge were recorded and analyzed [12].

Perioperative complications were categorized as minor and major complications as previously defined [13]. Major complications were defined as the complications that were life-threatening or may adversely affect the outcome of the treatment. Minor complications were defined as medical events noted in the medical records but did not compromise outcome.

### Radiographic measurements

For all patients, both anterior-posterior and lateral whole-spine X-rays were included to measure parameters. The following parameters, including sagittal vertical axis (SVA), thoracic kyphosis (TK), pelvic tilt (PT), sacral slope (SS), lumbar lordosis (LL), pelvic incidence (PI), and pelvic incidence minus lumbar lordosis (PI-LL), were recorded to assess the degree of spinal deformities in the coronal and sagittal plane. These data were measured on both the preoperative and the immediate postoperative radiographs. All measurements were performed independently by two spinal surgeons to decrease subjective bias.

### Statistical analysis

Data analysis was performed with SPSS version 25.0 (SPSS, Inc., Chicago, IL, USA). Continuous variables are reported as mean ± standard deviations (SD). Categorical variables are presented as a number or ratio. In the univariate testing, continuous variables were examined by the Student t-test. Categorical variables were tested by the Pearson chi-square test or Fisher’s exact test, depending on which was appropriate. Predictors with a *P* value < 0.2 on univariate analysis were included in the multivariate analysis.

The selection of variables in the final model was not only driven by statistical power but also by clinical judgement, collinearity, and previously reported risk factors. These variables were analyzed using a binary logistic regression model. Variables that differed significantly between those groups were then entered into a multivariate logistic regression analysis to identify independent risk factors. We generated a receiver operating characteristic (ROC) curve using predicted probability values from the logistic regression. Data were analyzed using SPSS software 20.0 (Chicago, Illinois, USA).

## Result

### Baseline characteristics and surgical characteristics

A total of 146 patients were included in our study. The preoperative assessment showed that 33.5% of the patients in our cohort had mild or severe systemic disease (ASA 2–3). The lowest instrumented vertebra of most cases was at L4 or upper (121 patients, 82.8%). Instrumented vertebral levels, ranging from 4 to 16, could be divided into two groups: 4 to 10 (43 patients, 29.4%) and 11 to 16 (103 patients, 66.5%). Fusion levels, ranging from 4 to 15, could be divided into two groups: 4 to 10 (60 patients, 41.1%) and 11 to 15 (86 patients, 58.9%). Decompression and osteotomy were performed in 2.1% (3 of 146) and 19.2% (28 of 146) of our patients, respectively. The radiographic parameters were based on anterior-posterior and lateral whole-spine X-rays. Prior to surgery, the average Cobb angle, SVA, TK, PT, SS, LL, PI, and PI -LL were as follows: 59.1 ± 19.7°, 23.2 ± 17.0 mm, 29.7 ± 15.7°, 9.6 ± 7.9°, 30.9 ± 12.7°, 46.4 ± 16.4°, 39.8 ± 16.1°, 12.9 ± 14.3°; the respective postoperative data were the following: 23.8 ± 17.2°, 19.4 ± 16.1 mm, 30.7 ± 11.3°, 10.3 ± 7.7°, 28.3 ± 12.5°, 43.8 ± 12.4°, 37.8 ± 16.9°, 8.1 ± 8.4°. A summary of baseline characteristics and surgical characteristics is shown in Tables 1 and 2.

**Table 1:** Demographics and baseline characteristics of included patients

Characteristics	Without perioperative complication (*n* = 125)	With perioperative complication (*n* = 21)	*P* value
Age (y/o)	28.8 ± 9.6	32.9 ± 10.7	0.081
Gender (male %)			0.850
Male	21(14.4%)	2(1.4%)	
Female	104(71.2%)	19(13.0%)	
BMI (kg/m^2^)	20.4 ± 2.8	20.7 ± 3.6	0.687
Symptom duration (month)	12.4 ± 9.3	15.5 ± 10.0	0.069
Smoking			1
No	120(82.2%)	20(13.7%)	
Yes	5(3.4%)	1(0.7%)	
Heart disease			0.717
No	121(82.9%)	20(13.7%)	
Yes	4(2.7%)	1(0.7%)	
Respiratory disease			1
No	116(79.5%)	20(13.7%)	
Yes	9(6.2%)	1(0.7%)	
Hypertension			0.346
No	123(84.2%)	20(13.7%)	
Yes	2(1.4%)	1(0.7%)	
Anemia			1
No	124 (84.9%)	21(14.4%)	
Yes	1(0.7%)	0(0.0%)	
History of surgery			
No	99(67.8%)	12(8.2%)	0.068
Yes	26(17.8%)	9(6.2%)	
ASA classification			0.048
1	87(59.6%)	10(6.8%)	
2–3	38(26.0%)	11(7.5%)	

*y/o* years old, *BMI* body mass index, *ASA* American Society of Anesthesiologists

**Table 2:** Operative characteristics of included patients

Characteristics	Without perioperative complication (n = 125)	With perioperative complication (n = 21)	*P* value
LIV			0.952
L4 or upper	103(70.5%)	18(12.3%)	
L5 or lower	22(15.1%)	3(2.1%)	
RBC transfusion			0.233
< 4u	106(72.6%)	15(10.3%)	
> =4u	19(13.0%)	6(4.2%)	
Operative time (min)	249.7 ± 73.4	260.9 ± 73.8	0.516
Length of hospital stay (day)	13.4 ± 4.0	16.9 ± 6.0	0.017
Estimated blood loss (mL)	689.9 ± 415.5	884.5 ± 554.6	0.061
Preoperative Hgb (g/L)	130.9 ± 15.1	133.1 ± 7.3	0.288
Preoperative albumin (g/L)	43.1 ± 3.9	42.6 ± 2.5	0.512
Fusion levels			0.026
4–10	56(38.4%)	4(2.7%)	
11–15	69(47.3%)	17(11.6%)	
Instrumented vertebral levels			0.258
4–10	39(26.7%)	4(2.7%)	
11–16	86(58.9%)	17(7.6%)	
Decompression			0.375
No	123(84.2%)	20(13.7%)	
Yes	2(1.4%)	1(0.7%)	
Spinal osteotomy			0.038
No	105(71.9%)	13(8.9%)	
Yes	20(13.7%)	8(5.5%)	
Cobb (°)			
Preoperative	59.1 ± 19.7	73.1 ± 21.9	0.004
Postoperative	23.8 ± 17.2	28.3 ± 19.4	0.274
Change	35.3 ± 10.2	44.8 ± 17.0	0.022
SVA (mm)			
Preoperative	23.2 ± 17.0	20.3 ± 20.8	0.486
Postoperative	19.4 ± 16.1	17.7 ± 20.9	0.663
Change	15.0 ± 13.7	19.6 ± 19.5	0.315
TK (°)			
Preoperative	29.7 ± 15.7	36.3 ± 18.3	0.086
Postoperative	30.7 ± 11.3	35.7 ± 13.4	0.076
Change	10.4 ± 9.7	10.1 ± 7.3	0.899
PT (°)			
Preoperative	9.6 ± 7.9	9.0 ± 7.8	0.734
Postoperative	10.3 ± 7.7	8.8 ± 8.3	0.413
Change	5.4 ± 4.6	5.5 ± 5.1	0.898
SS (°)			
Preoperative	30.9 ± 12.7	31.3 ± 14.9	0.900
Postoperative	28.3 ± 12.5	27.9 ± 15.5	0.894
Change	7.9 ± 10.8	10.4 ± 10.0	0.314
LL (°)			
Preoperative	46.4 ± 16.4	49.0 ± 14.9	0.492
Postoperative	43.8 ± 12.4	49.8 ± 16.1	0.050
Change	10.0 ± 8.0	11.8 ± 7.9	0.354
PI (°)			
Preoperative	39.8 ± 16.1	38.8 ± 20.1	0.810
Postoperative	37.8 ± 16.9	34.8 ± 21.0	0.456
Change	9.1 ± 12.9	9.6 ± 13.9	0.877
PI-LL (°)			
Preoperative	12.9 ± 14.3	16.7 ± 15.3	0.246
Postoperative	8.1 ± 8.4	11.1 ± 11.2	0.143
Change	4.7 ± 13.0	3.7 ± 12.0	0.256

*LIV* lowest instrumented vertebra, *RBC* red blood cell, *SVA* sagittal vertical axis, *TK* thoracic kyphosis, *PT* pelvic tilt, *SS* sacral slope, *LL* lumbar lordosis, *PI* pelvic incidence, *PI –LL* pelvic incidence minus lumbar lordosis

### Perioperative complications

Of 146 patients, there were 21 (14.4%) patients who suffered from complications during the perioperative periods, including eight (34.8%) cardiopulmonary complications, two (8.7%) infection complications, six (26.1%) neurological complications, three (13.0%) gastrointestinal complications, and four (17.4%) incision-related complications. Eighteen patients (12.3%) suffered one complication. Two complications were recorded in each of 2 patients (1.4%) (Table 3).

**Table 3:** Distribution of perioperative complications

Type	Major perioperative complication	Number	Minor perioperative complication	Number of patients	Total
Cardiopulmonary	Congestive heart failure	1 (4.3%)	Atelectasis	1 (4.3%)	8 (34.8%)
	Pleural effusion	6 (26.1%)			
Infection			Urinary infection	2 (8.7%)	2 (8.7%)
Neurological	Nerve root injury	1 (4.3%)	Radicular edema	2 (8.7%)	6 (26.1%)
			Sensory deficit	3 (13.0%)	
Gastrointestinal	Acute pancreatitis	1 (4.3%)	Ileus	2 (8.7%)	3 (13.0%)
Incision-related	Fat liquefaction	2 (8.7%)	Non-aligned edges	1 (4.3%)	4 (17.4%)
			Atopic dermatitis	1 (4.3%)	

Different strategies were employed to deal with perioperative complications (Table 4). Closed thoracic drainage proved beneficial for patients with pleural effusion and atelectasis. To treat congestive heart failure, the patient’s fluid status was closely monitored, and drugs were given to promote diuresis. Urinary infection was treated with urine culture and anti-infection drug. Acute neurological problems could be rapidly corrected by dehydration and steroid treatment, while chronic neurological symptoms required patience and a nerve-nurturing treatment. Generally, all symptoms tended to improve in the follow-up period.

**Table 4:** Category, management and treatment outcomes of perioperative complications

Category	Symptoms and signs	Management	Results
Cardiopulmonary	Pleural effusion, atelectasis	Thoracic closed drainage	Recovered in 2w postoperatively
	Congestive heart failure	Consultation with cardiology, myocardium-nurturing, and control fluid infusion	Recovered in 1w postoperatively
Infection	Urinary infection	Urine culture, anti-infection drug	Recovered in 2w postoperatively
Neurological	Radicular edema	Dehydration and steroid treatment	Recovered
	Sensory deficit, peripheral nerve palsy	Dehydration and nerve-nurturing treatment	Recovered in 4w postoperatively
	Nerve root injury	Revision surgery	Recovered
Gastrointestinal	Ileus	Fasting and water deprivation, acid-suppressive drugs, liquid paraffin, and glycerine enema	Recovered in 2w
	Acute pancreatitis	Fluid replacement, monitoring electrolytes and oxygen saturation, anti-infection, and acid-suppressive drugs	Recovered in 2w postoperatively
Incision	Fat liquefaction	Debridement and suture	Recovered in 1w postoperatively
	Non-aligned edges	Dressing change and anti-infection drugs	Recovered in 1w postoperatively
	Atopic dermatitis	Maintaining skin hydration and topical anti-inflammatory therapy	Recovered

### Univariate analysis

Patients were divided into two groups based on whether they had any perioperative complications: without perioperative complication group (125) and with perioperative complications group (21). The results of univariate analysis investigating the relationships between baseline/surgical characteristics and perioperative complications are shown in Tables 1 and 2. Factors that were found to carry a statistically significant weight in risk prediction were ASA classification (*P* = 0.048), LOS (*P* = 0.017), EBL (*P* = 0.061), levels of fusion (*P* = 0.026), spinal osteotomy (*P* = 0.038), preoperative Cobb angle (*P* = 0.004), change in Cobb angle (*P* = 0.022) and postoperative LL (*P* = 0.050). Predictors with *P* values < 0.2 were also considered eligible to be factored into risk calculations.

### Multivariate analysis

**Fig. 1:**
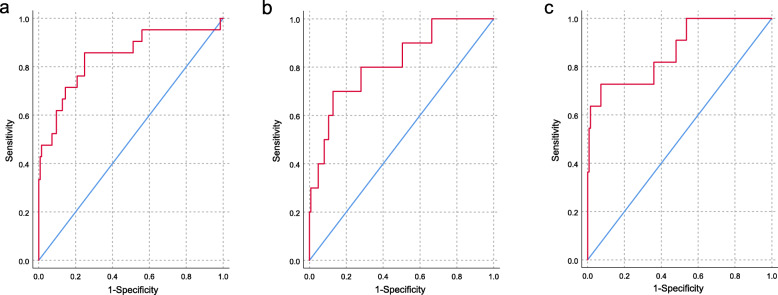
The ROC curve using predicted probability values from the logistic regression. **a** ROC curve of risk factors predicting total perioperative complications, **b** ROC curve of risk factors predicting minor perioperative complications, **c** ROC curve of risk factors predicting major perioperative complications

Factors whose *P* value < 0.2 in the univariate analysis were selected for multivariate analysis. The independent risk factors for development of total perioperative complications included change in Cobb angle (OR = 1.085, 95% CI = 1.035 ~ 1.137, *P* = 0.001) and spinal osteotomy (OR = 3.565, 95% CI = 1.039 ~ 12.236, *P* = 0.043). The area under the ROC curve based on predicted probability of the logistic regression was 0.842 (95% CI = 0.734 ~ 0.949) (Fig. 1a). The independent risk factor for minor perioperative complications is change in Cobb angle (OR = 1.092, 95% CI = 1.023 ~ 1.165, *P* = 0.008). The area under the ROC curve based on predicted probability of the logistic regression was 0.818 (95% CI = 0.678 ~ 0.959) (Fig. 1b). The independent risk factors for major perioperative complications are spinal osteotomy (OR = 4.475, 95% CI = 1.960 ~ 20.861, *P* = 0.036) and change in Cobb angle (OR = 1.106, 95% CI = 1.035 ~ 1.182, *P* = 0.003). The area under the ROC curve based on predicted probability of the logistic regression was 0.856 (95% CI = 0.742 ~ 0.989) (Fig. 1c). A summary of multivariate analysis results is shown in Table 5.

**Table 5:** Multivariate analysis of risk factors of perioperative complications

Complication	Variables	OR	95% CI	*P* value
Total complication	Spinal osteotomy	3.565	1.039, 12.236	0.043
	Cobb angle change	1.085	1.035, 1.137	0.001
Minor complication	Cobb angle change	1.092	1.023, 1.165	0.008
Major complication	Spinal osteotomy	4.475	1.960, 20.861	0.036
	Cobb angle change	1.106	1.035, 1.182	0.003

*OR* odds ratio, *CI* confidence interval, *LL* lumbar lordosis, *ASA* American Society of Anesthesiologists

## Discussion

Non-degenerative factors are presumed to account for as high as 90% of spinal deformities in young adults [10]. ASD patients often undergo long-segment thoracolumbar arthrodesis that extends to the lower lumbar spine or the sacral region, and this procedure is associated with more perioperative complications [6]. The treatment for adult non-degenerative scoliosis aims not only to prevent further progression but also seeks to improve the existing manifestations [14]. In this study, we collected a cohort of data on the perioperative complications after surgical treatment of adult non-degenerative scoliosis. The results of multivariate analysis reveal that the change in Cobb angle and spinal osteotomy are independent risk factors for the development of perioperative complications, the change in Cobb angle is an independent risk factor for the development of minor perioperative complications, the change in Cobb angle and spinal osteotomy are independent risk factor for the development of major perioperative complications.

Surgical treatment is recommended when conservative treatment proves unsatisfactory, and decompression surgery is essential for alleviating symptoms. Most surgeons recommend fusion and instrumentation techniques for decompression [15]. Thus, choosing the proper extent of the fusion is key to a successful surgery. Long fusion and instrumentation proved successful in correcting scoliotic curvature and coronal imbalance. For patients with a large Cobb angle and rotatory subluxation, long fusion should be carried out to minimize adjacent segment disease [6]. All the patients selected for our study had long fusions, and their levels of distal fusions were different. Stopping a fusion at L5 can lead to subsequent degeneration at L5-S1. If the fusion extends to the sacrum, the procedure would be more complex, and there is a higher likelihood of pseudarthrosis at the lumbosacral junction. However, studies have found that long fusions terminating at L5 or the sacrum was similar in overall complication rate and improvement in pain and disability [16, 17]. In our study, we found no association between the incidence of perioperative complications and the level at which the fusion stopped (*P* = 0.952). There is a new instrument method, the S2AI iliac screw, which is designed to fix drawbacks such as screw site prominence and wound complication, that can successfully avoid the complications associated with conventional iliac screws [18]. However, this presumed reduction in perioperative complications in the S2AI group was not detected by our study, which might be due to our limited sample size.

Focused on adult non-degenerative scoliosis patients who underwent long fusion surgeries, we collected and analyzed all the parameters deemed relevant according to our clinical expertise and previous research, which involved collecting the patients’ medical history, radiographic data, and clinical evaluations. Owing to the fact that most of our patients were relatively young, there was relatively little data on history of lumbar operation, previous medication use, or whether there were any age-associated comorbidities such as diabetes and osteoporosis, some of which could be potential risk factors for ADS. Several studies have reported a direct correlation between parameters such as the ASA grade, Cobb angle, total operation time, PT, level of fusion, LOS, staging, multiple surgeries and the incidence of perioperative complications in ADS [9, 19–22]. However, further research is needed to identify the risk factors for perioperative complications in adult non-degenerative scoliosis.

In this study, change of main Cobb angle were significantly associated with the risk of minor and major perioperative complications. We included changes of all parameters in our analysis, which have rarely been reported in previous studies. Previous studies have shown that preoperative magnitude of the spinal curvature and coronal imbalance was associated with the likelihood of complications. Some author reported that an increased Cobb angle is associated with impaired pulmonary function due to airway blockage [23]. A higher risk of postoperative non-neurological complications, pulmonary compromise in particular, could be caused by a larger Cobb angle in adults and juvenile scoliosis patients [24]. An increased Cobb angle causes abnormal chest and lung development and results in less reserved space for ventilation.

Osteotomy is an effective procedure to correct spinal deformity, but it is often accompanied by some complications. In the Sciubba’s study, they found that the most common complication after three-column osteotomies was neurological deficits [25]. In the Buchowski’s study, they reported that the incidence of neurological complications was 11.1% after lumbar pedicle subtraction osteotomies (PSO) [26]. In our study, osteotomy procedure was significantly associated with the risk of total perioperative complications (*P* = 0.043, OR = 3.565, 95% CI = 1.039, 12.236) and major perioperative complications (*P* = 0.036, OR = 4.475, 95% CI = 1.960, 20.861). We consider that osteotomy procedure is usually associated with large surgical injury, which not only causes a high incidence of nerve injury, but also leads to abnormal homeostasis and stress states in patients. The combined effect of these factors may lead to the occurrence of complications.

There are some limitations to our study. First, the most significant being its retrospective nature. Second, in this study, all of the data was obtained from single medical center, and results were not validated by other centers. Third, due to the relatively young age of many of our patients, the effect of comorbidities that are more prevalent in elderly populations could not be adequately investigated.

In summary, we observed a 21 of 146 patients experienced complications during the perioperative periods. The change in Cobb angle and spinal osteotomy may contribute to the development of perioperative complications. The identification of these risk factors has potential to help stratify preoperative risks and reduce the incidence of complications.

## Data Availability

All of the patient’s medical record and images are kept in Peking Union Medical College Hospital. For the review, please refer to the method section.

## Abbreviations

OR: odds ratio
RBC: red blood cell
ROC: receiver operating characteristic
ASD: adult spinal deformities
ADS: adult degenerative scoliosis
BMI: body mass index
Hgb: hemoglobin
ASA: American Society of Anesthesiologists
SVA: sagittal vertical axis
TK: thoracic kyphosis
PT: pelvic tilt
SS: sacral slope
LL: lumbar lordosis
PI: pelvic incidence
PI-LL: pelvic incidence minus lumbar lordosis
LOS: length of hospital stays
AIS: adolescent idiopathic scoliosis
